# Early Recognition and Management of Arrhythmogenic Right Ventricular Cardiomyopathy in a Young Athlete: A Case Report Highlighting the Role of Multimodal Diagnosis and Preventive Implantable Cardioverter Defibrillator (ICD) Therapy

**DOI:** 10.7759/cureus.90736

**Published:** 2025-08-22

**Authors:** Brittni McClellan, Dhruva Govil, Andrew Sherman, Christopher Bradley

**Affiliations:** 1 Cardiology, Henry Ford Health System, Southfield, USA; 2 Internal Medicine, Henry Ford Health System, Southfield, USA

**Keywords:** arrhythmogenic right ventricular cardiomyopathy (arvc), implantable cardioverter-defibrillator (icd), plakophilin-2 (pkp2) mutation, sudden cardiac death (scd), ventricular tachycardia (vt)

## Abstract

Arrhythmogenic Right Ventricular Cardiomyopathy (ARVC) is a rare inherited cardiomyopathy marked by fibrofatty replacement of right ventricular (RV) myocardium, leading to electrical instability and increased risk for ventricular arrhythmias and sudden cardiac death (SCD). ARVC is typically inherited in an autosomal dominant pattern and often involves mutations in desmosomal proteins such as plakophilin-2 (PKP2). Clinical presentation can vary from asymptomatic to life-threatening arrhythmias, particularly among young athletes. This case emphasizes the importance of early detection and intervention in patients with ARVC.

A 21-year-old previously healthy male presented with recurrent exertional syncope. Initial ECG demonstrated sinus rhythm with T-wave inversions in leads V1-V3. Coronary CT angiography and resting echocardiography were normal. However, the stress electrocardiogram revealed frequent premature ventricular complexes (PVCs) with a left bundle branch block morphology. Ambulatory event monitoring detected over 500 monomorphic PVCs in a 24-hour period. Cardiac MRI demonstrated mildly reduced RVEF (39%) with regional dyskinesia, meeting major diagnostic criteria per the 2010 Task Force Criteria for ARVC. Genetic testing confirmed a heterozygous pathogenic mutation (c.1689-1G>C) in the PKP2 gene.

The patient was started on beta-blockers and underwent implantation of a single-chamber implantable cardioverter defibrillator (ICD) due to elevated arrhythmic risk. Post-implantation, the ICD successfully terminated three episodes of ventricular tachycardia, highlighting its life-saving role. This case underscores the necessity for a high index of suspicion when evaluating young patients with unexplained syncope or arrhythmias. Diagnosis of ARVC requires integration of clinical, electrocardiographic, imaging, and genetic data. Early diagnosis and prompt management, including activity modification, pharmacologic therapy, and ICD implantation, are critical to mitigating the risk of SCD. Genetic testing and vigilant follow-up remain essential components in the care of ARVC patients.

## Introduction

Arrhythmogenic Right Ventricular Cardiomyopathy (ARVC) is a rare, inherited condition marked by fibrofatty replacement of the right ventricular (RV) myocardium. This structural remodeling impairs electrical conduction and increases the risk of ventricular arrhythmias and sudden cardiac death (SCD) [1]. Although considered rare, ARVC is estimated to affect between one in 1,000 and one in 5,000 individuals, though true prevalence may be underestimated due to variable expression and incomplete penetrance [2]. It is most commonly inherited in an autosomal dominant fashion. ARVC is frequently linked to mutations in desmosomal proteins, particularly plakophilin-2 (PKP2). Desmosomes are intercellular junctions that anchor adjacent cardiomyocytes and maintain the mechanical and electrical integrity of the heart during contraction. Disruption of these proteins weakens myocardial cohesion and sets the stage for arrhythmogenesis.

Clinical presentation varies widely, from asymptomatic individuals to those experiencing syncope, sustained arrhythmias, or SCD, especially in young athletes. Diagnosis relies on a combination of clinical, imaging, electrocardiographic, histopathologic, and genetic criteria, as outlined in the Revised 2010 Task Force Guidelines. Given this variability, a strong family history of ARVC or unexplained sudden death should raise clinical suspicion and prompt early screening [3]. Given the often unpredictable nature of disease progression, implantable cardioverter-defibrillators (ICDs) are commonly used as a preventive strategy in high-risk patients. This case underscores the importance of early recognition, genetic evaluation, and timely intervention in altering the disease course.

## Case presentation

A 21-year-old previously healthy male presented with recurrent exertional syncope. Patient stated that over the last month, he had two different episodes of syncope, which occurred only with exertion, and he did not experience any preceding symptoms. He stated that his episodes would last for roughly 30 seconds to 1 minute in length and would then wake up with no post-ictal state. During these episodes, he denies any tongue biting or incontinence. Upon further investigation, he denied any family history of any cardiac diseases other than coronary artery disease (CAD).

**Figure 1:**
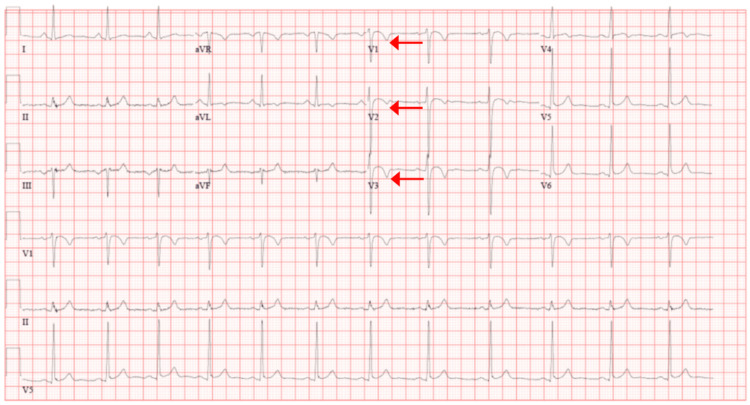
Initial electrocardiogram (ECG) Findings of sinus rhythm with T-wave inversions in the right precordial leads, extending from V1-V3.

Upon arrival, the patient was hemodynamically stable and normotensive, afebrile, with a heart rate of 93 beats per minute. Laboratory tests, especially troponin and thyroid-stimulating hormone, were negative. Initial electrocardiogram (ECG) revealed normal sinus rhythm with normal axis with evidence of left ventricular hypertrophy (LVH), as indicated by increased QRS voltage and a repolarization pattern in the lateral leads. T-wave inversions are noted in leads V1-V3, raising suspicion for an underlying arrhythmogenic process (Figure 1).

**Figure 2:**
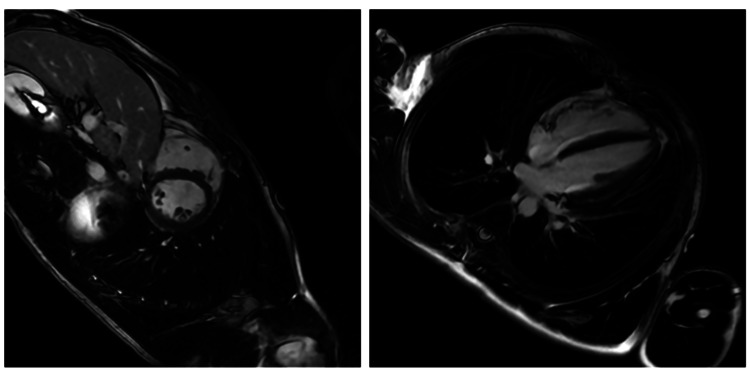
Cardiac MRI Left: Short-axis view at mid-ventricular level shows no abnormal late gadolinium enhancement in the myocardium. Right: Long-axis 4-chamber view shows no abnormal late gadolinium enhancement. (Images were obtained following injection of 26mL intravenous (IV) contrast and after T1 suppression).

**Figure 3:**
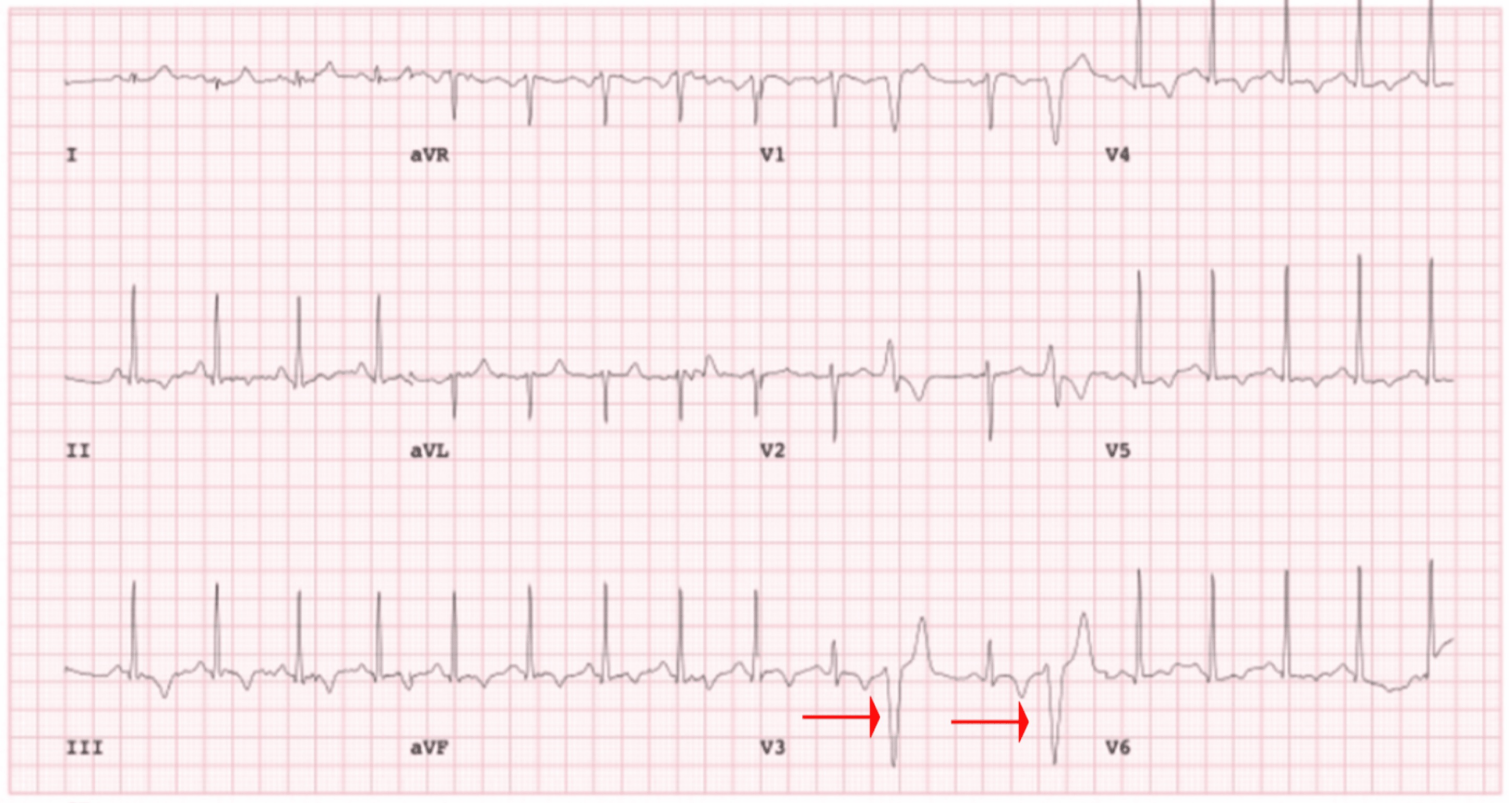
Stress test ECG The electrocardiogram (ECG) revealed multiple premature ventricular complexes (PVCs) (seen by red arrows).

Coronary CT angiography and resting echocardiography were unremarkable, ruling out coronary disease and overt structural abnormalities. A stress ECG showed frequent premature ventricular complexes (PVCs) with a left bundle branch block morphology, suggestive of RV origin. The patient was discharged with a wearable cardiac monitor and advised to restrict physical activity. Prior to genetic testing, the patient had a borderline diagnosis of ARVC based on the 2010 Task Force Criteria [4]. He met one major criterion: cardiac magnetic resonance imaging (CMR) demonstrated mildly reduced right ventricular ejection fraction (RVEF) at 39% with regional dyskinesia, though without late gadolinium enhancement (LGE) (Figure 2). While LGE supports the diagnosis by indicating myocardial fibrosis, its absence does not exclude ARVC in earlier or less fibrotic stages of the disease. He also met one minor criterion: ambulatory event monitoring revealed over 500 monomorphic premature ventricular complexes (PVCs) within a 24-hour period, supporting the presence of an arrhythmic substrate. This was seen on the stress ECG, which revealed sinus rhythm with a wide QRS complex consistent with left bundle branch block (LBBB), along with multiple premature ventricular complexes (PVCs) (Figure 3).

**Figure 4:**
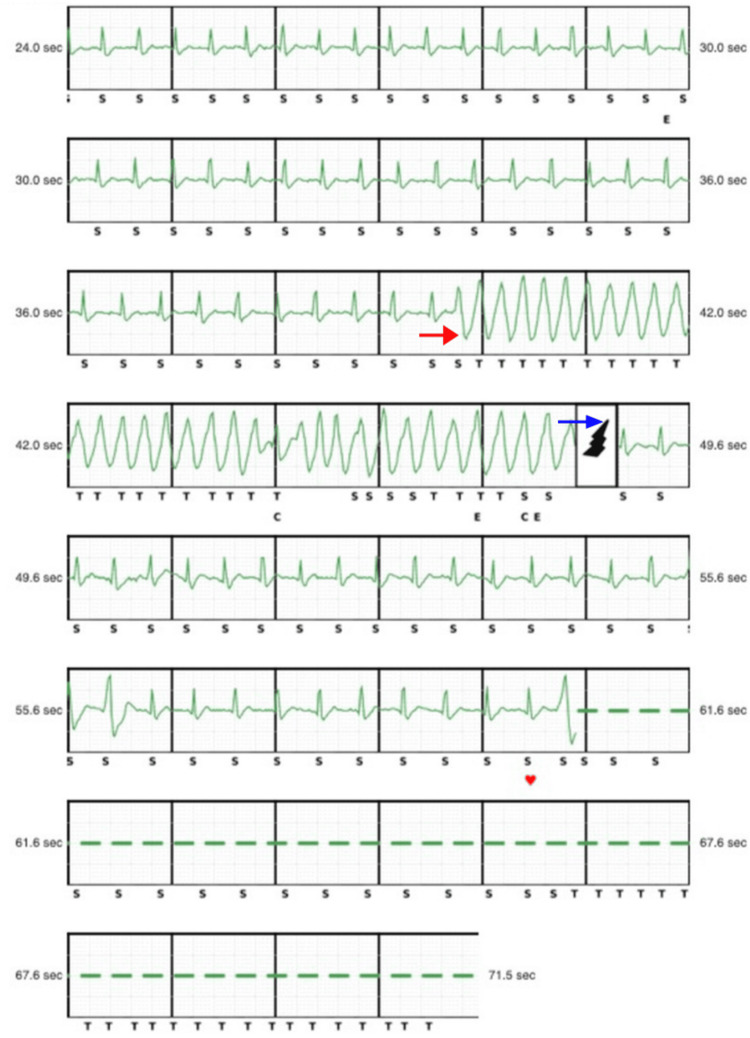
Implantable cardioverter-defibrillator (ICD) interpretation The figure highlights one of the three episodes of ventricular tachycardia as seen by the red arrow. It also demonstrates that the device successfully terminated the arrhythmia via shock delivery, as seen by the blue arrow.

These criteria, along with his symptomatology, resulted in beta-blocker initiation, and genetic testing revealed a heterozygous pathogenic c.1689-1G>C mutation in the PKP2 gene, confirming the diagnosis. Due to elevated risk for SCD, a single-chamber implantable cardioverter-defibrillator (ICD) was placed. Post-implant, the patient experienced three episodes of ventricular tachycardia (VT), all terminated by appropriate ICD therapy (Figure 4). Post-ICD intervention, the patient returned to normal sinus rhythm.

## Discussion

**Figure 5:**
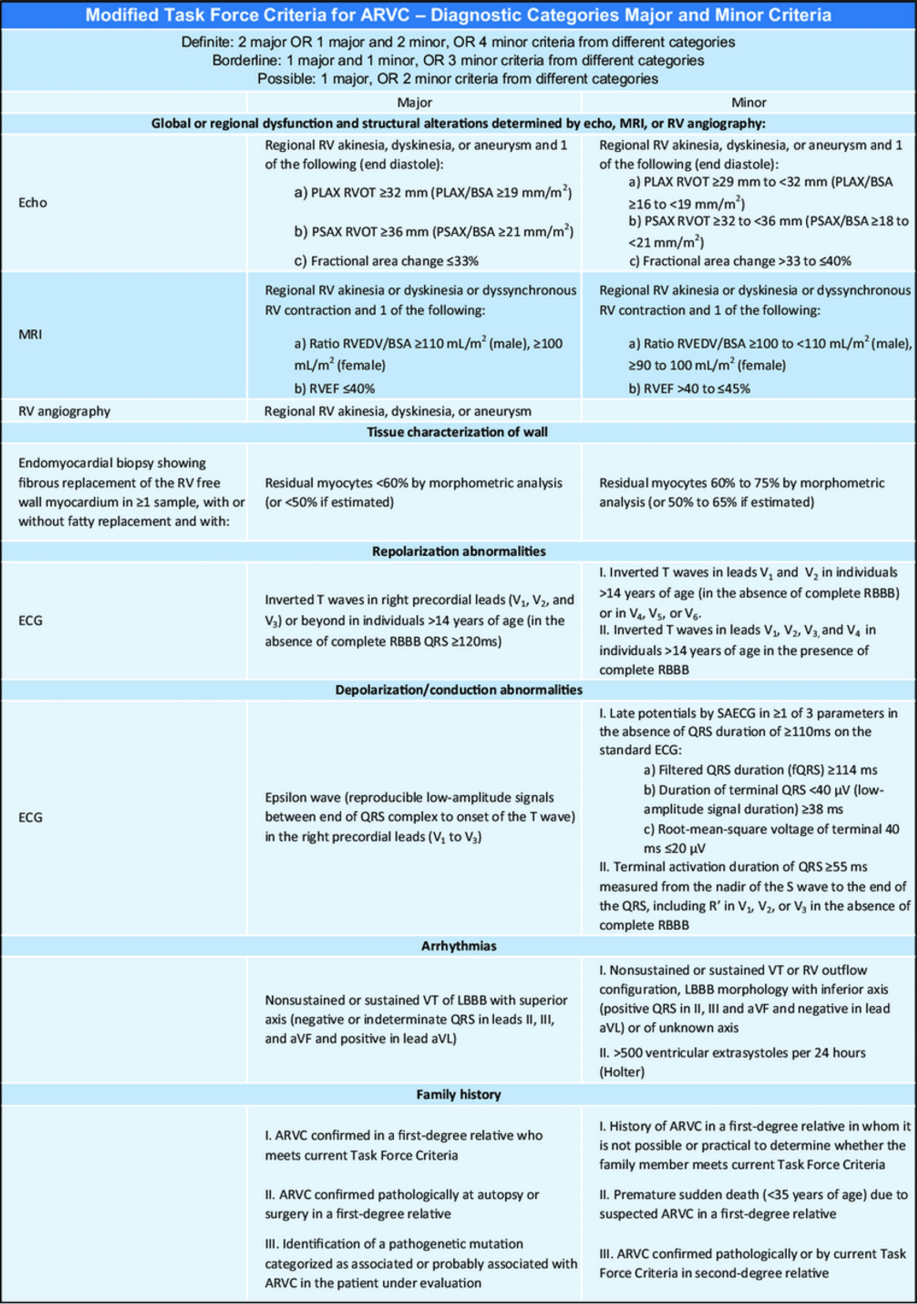
Revised task force criteria The figure shows the 2010 revised Task Force criteria for the diagnosis of arrhythmogenic right ventricular cardiomyopathy (ARVC). Image from ResearchGate [5].

This case underscores the critical importance of maintaining a high index of suspicion for ARVC in young individuals presenting with unexplained arrhythmic events or syncope, particularly when ECG abnormalities or symptoms suggestive of arrhythmia are present. Diagnosis of ARVC typically requires a combination of clinical, electrocardiographic, imaging, and genetic assessments [4]. According to the 2010 Task Force Criteria, the diagnosis is based on a combination of major and minor criteria in several categories, including the presence of arrhythmias, structural abnormalities, regional ventricular dysfunction, and family history, in addition to molecular genetic findings (Figure 5).

In this case, the patient met two major criteria: (1) the presence of a pathogenic PKP2 mutation, which is classified as pathogenic per American College of Medical Genetics and Genomics (ACMG) guidelines, and (2) RV dyskinesia in combination with reduced ejection fraction (EF) =<40%. According to cardiac MRI (CMR) criteria, a major structural abnormality is defined as regional RV akinesia, dyskinesia, or dyssynchronous contraction and either an RV EF =<40% or an indexed RV end-diastolic volume ≥110 mL/m^2^ (male) or ≥100 mL/m^2^ (female). The patient also met a minor criterion via the Holter monitoring/ECG, which revealed a PVC burden of 18% (>500 PVC) over 24 hours, supporting the diagnosis and aiding in risk stratification [4,6,7].

Management of ARVC is multifaceted, with the primary goals being the prevention of arrhythmias, symptom control, and the reduction of the risk of sudden cardiac death. This typically involves a combination of medical therapy, including beta-blockers and antiarrhythmic drugs, along with lifestyle modifications such as activity restriction. The patient was initiated on metoprolol succinate 50 mg daily, which he tolerated without significant bradycardia or hypotension. Due to persistent PVCs and concern for disease progression, flecainide was later added at a dose of 50 mg twice a day (BID) under close monitoring. In high-risk patients, like those with a history of cardiac arrest, severe RV dysfunction, or LV dysfunction, ICD implantation is indicated for secondary prevention of sudden cardiac arrest [6,8,9]. Although this patient did not experience an episode of sustained VT prior to ICD implantation, the decision to proceed with device placement was based on the physician’s strong clinical suspicion of ARVC, genetic testing results, and the presence of frequent PVCs. 

Upon follow-up, the patient remained hemodynamically stable. ICD interrogation over the subsequent nine-month follow-up revealed three episodes of non-sustained ventricular tachycardia (NSVT), which resolved with subsequent shocks. At the last follow-up, 12 months post-ICD placement, the patient remained hemodynamically stable and had not experienced syncope or ICD shocks. Repeat echocardiogram showed preserved biventricular function with stable RV dimensions compared to baseline. He continued to adhere to lifestyle recommendations, including avoiding vigorous physical activity, which is a key component of secondary prevention in ARVC patients [10].

Given the progressive and unpredictable nature of ARVC, lifelong follow-up is essential. Regular monitoring helps detect early signs of worsening heart function or new arrhythmias before complications arise. Follow-up is typically recommended every 1-2 years, depending on age, symptoms, or disease severity. These visits usually include clinical history, ECG, echocardiogram, wearable monitoring, and sometimes exercise testing to detect effort-induced ventricular arrhythmias. Ongoing structured care is key to helping patients manage ARVC safely and maintain their quality of life over time.

## Conclusions

This case highlights the importance of early recognition and intervention in patients with ARVC, a condition that, although rare, should remain on the differential diagnosis for young individuals presenting with unexplained arrhythmias, syncope, or other related symptoms. Clinicians must be vigilant in identifying the subtle signs and symptoms that could indicate ARVC, as early diagnosis and appropriate management can significantly reduce the risk of serious complications, such as ventricular arrhythmias and sudden cardiac death. This case also underscores the importance of genetic testing and the preventive benefit of ICD placement in high-risk patients, along with long-term monitoring. While this case offers valuable insights, the findings and implications should be interpreted cautiously, as broader validation through larger patient cohorts is necessary to confirm generalizability.

## References

[1] Hlatky MA, Greenland P, Arnett DK (2009). Criteria for evaluation of novel markers of cardiovascular risk: a scientific statement from the American Heart Association. Circulation.

[2] Marcus FI, Fontaine GH, Guiraudon G, Frank R, Laurenceau JL, Malergue C, Grosgogeat Y (1982). Right ventricular dysplasia: a report of 24 adult cases. Circulation.

[3] Corrado D, Fontaine G, Marcus FI, McKenna WJ, Nava A, Thiene G, Wichter T (2000). Arrhythmogenic right ventricular dysplasia/cardiomyopathy: need for an international registry. Circulation.

[4] Komajda M, Weidinger F, Kerneis M (2016). EURObservational Research Programme: the chronic ischaemic cardiovascular disease registry: pilot phase (CICD-PILOT). Eur Heart J.

[5] Towbin JA, McKenna WJ, Abrams DJ (2019). 2019 HRS expert consensus statement on evaluation, risk stratification, and management of arrhythmogenic cardiomyopathy: executive summary. Heart Rhythm.

[6] Krahn AD, Wilde AA, Calkins H, La Gerche A, Cadrin-Tourigny J, Roberts JD, Han HC (2022). Arrhythmogenic right ventricular cardiomyopathy. JACC Clin Electrophysiol.

[7] Corrado D, Basso C, Thiene G (2000). Arrhythmogenic right ventricular cardiomyopathy: diagnosis, prognosis, and treatment. Heart.

[8] Towbin JA, McKenna WJ, Abrams DJ (2019). 2019 HRS expert consensus statement on evaluation, risk stratification, and management of arrhythmogenic cardiomyopathy. Heart Rhythm.

[9] Wang W, James CA, Calkins H (2019). Diagnostic and therapeutic strategies for arrhythmogenic right ventricular dysplasia/cardiomyopathy patient. Europace.

[10] James CA, Bhonsale A, Tichnell C (2013). Exercise increases age-related penetrance and arrhythmic risk in arrhythmogenic right ventricular dysplasia/cardiomyopathy-associated desmosomal mutation carriers. J Am Coll Cardiol.

